# Connectivity modulations induced by reach&grasp movements: a multidimensional approach

**DOI:** 10.1038/s41598-021-02458-x

**Published:** 2021-11-29

**Authors:** Pietro Caliandro, Gloria Menegaz, Chiara Iacovelli, Carmela Conte, Giuseppe Reale, Paolo Calabresi, Silvia F. Storti

**Affiliations:** 1 UOC Neurologia - Dipartimento Scienze dell’Invecchiamento, Neurologiche, Ortopediche e della Testa-Collo, Fondazione Policlinico Universitario A. Gemelli IRCCS, Rome, Italy; 2 Department of Computer Science, University of Verona, Strada Le Grazie 15, 37134 Verona, Italy; 3 UOC Riabilitazione e Medicina Fisica - Dipartimento Scienze dell’Invecchiamento, Neurologiche, Ortopediche e della Testa-Collo, Fondazione Policlinico Universitario A. Gemelli IRCCS, Largo Agostino Gemelli 8, 00168 Rome, Italy; 4 Policlinico Italia, Piazza del campidano 6, 00162 Roma, Italy; 5 UOC Neuroriabilitazione ad Alta Intensità - Dipartimento Scienze dell’Invecchiamento, Neurologiche, Ortopediche e della Testa-Collo, Fondazione Policlinico Universitario A. Gemelli IRCCS, Rome, Italy; 6 Clinica Neurologica, Dipartimento di Neuroscienze, Fondazione Policlinico Universitario Agostino Gemelli IRCCS, Università Cattolica del Sacro Cuore, Rome, Italy

**Keywords:** Neuroscience, Neurology, Engineering

## Abstract

Reach&grasp requires highly coordinated activation of different brain areas. We investigated whether reach&grasp kinematics is associated to EEG-based networks changes. We enrolled 10 healthy subjects. We analyzed the reach&grasp kinematics of 15 reach&grasp movements performed with each upper limb. Simultaneously, we obtained a 64-channel EEG, synchronized with the reach&grasp movement time points. We elaborated EEG signals with EEGLAB 12 in order to obtain event related synchronization/desynchronization (ERS/ERD) and lagged linear coherence between Brodmann areas. Finally, we evaluated network topology via sLORETA software, measuring network local and global efficiency (clustering and path length) and the overall balance (small-worldness). We observed a widespread ERD in α and β bands during reach&grasp, especially in the centro-parietal regions of the hemisphere contralateral to the movement. Regarding functional connectivity, we observed an α lagged linear coherence reduction among Brodmann areas contralateral to the arm involved in the reach&grasp movement. Interestingly, left arm movement determined widespread changes of α lagged linear coherence, specifically among right occipital regions, insular cortex and somatosensory cortex, while the right arm movement exerted a restricted contralateral sensory-motor cortex modulation. Finally, no change between rest and movement was found for clustering, path length and small-worldness. Through a synchronized acquisition, we explored the cortical correlates of the reach&grasp movement. Despite EEG perturbations, suggesting that the non-dominant reach&grasp network has a complex architecture probably linked to the necessity of a higher visual control, the pivotal topological measures of network local and global efficiency remained unaffected.

## Introduction

The Reach&grasp movement has a pivotal ethological meaning and represents an everyday action which implies a strong functional interaction among cerebral areas^1–3^. Acute stroke alters brain networking^4,5^ and may cause severe disability with impairment of upper limb function^6,7^. After a stroke, brain plasticity can promote or hinder motor recovery^8,9^. In this framework, an integrated method able to simultaneously measure brain connectivity and kinematics of a finalistic and spontaneous movement as reach&grasp, could possibly allow to evaluate the upper limb kinematic features and pathophysiological mechanisms during the recovery process. However, an integrated method able to simultaneously measure brain connectivity and kinematics of the reach&grasp movement is not yet available for humans. Traditionally, two different cortical anatomical pathways encode the reach&grasp movement as part of a dual-channels hypothesis^10,11^. Specifically, the anterior intraparietal sulcus (AIP), an area enclosed in the posterior parietal cortex, elaborates visual and sensory information regarding the position and features of the object to be grasped^12^ and guides the function of the dorsal and ventral premotor cortex (respectively PMd and PMv) in the reach&grasp execution. PMd and PMv would be part of two anatomically different but functionally coordinated networks, respectively a dorsomedial parietofrontal pathway encoding reaching movements of the arm^13^ and a dorsolateral parietofrontal network for grasping movements of the hand^2,14^. Recently, the dual-channels hypothesis has been reappraised, in light of the evidence that both PMd and PMv might encode the kinematics of reaching and grasping movements, suggesting that PMd and PMv are not functionally segregated in macaques^15,16^ as in humans^17^. In other words, different cortical areas, namely PMd and PMv, seem to be functionally active in either the reaching or grasping components of the motor task, suggesting a more complex cortical representation^15,16^. Some evidence demonstrates that finger and hand tasks, as well as flexo-extension tasks with upper limbs, modulate cortical desynchronization mainly in α (8–12 Hz) and β (13–30 Hz) frequency bands^18,19^ and determine changes of functional networking^20,21^. Finally, it has been pointed out that reach&grasp tasks might somehow modulate functional connectivity (FC) between cortical areas^22^. The brain connectome is a functionally interconnected network that assures rapid segregation and integration of information processing. In this context, graph theory analyses functional brain networks and describes their topological properties, becoming an essential tool to quantify the physiological and pathological patterns in neuroscience studies^23^. A graph is defined as a set of nodes (brain regions) interconnected by a set of edges (connections). FC is usually expressed in connectivity matrices, from which network topology can be derived at either local (functional segregation) or global (functional integration) levels through several properties. Moreover, studying the small-world organization provides insight to the balance between functional segregation, on one side, and global integration capacity on the other side^24–26^.

Our working hypothesis is that the reach&grasp kinematics is associated to functional connectivity changes specific for the different EEG frequency bands in healthy subjects. Moreover, we hypothesize that the reach&grasp related FC changes do not alter the cortex small-world architecture, in order to maintain the network efficiency and to satisfy its parallel and integrated needs of local and global processing.

## Results

### Kinematics

The jerk evaluation showed no statistical difference between right and left arm (the jerk values were 703.63 ± 183.39 and 641.86 ± 194.54 respectively for the right and the left side). Also kinematic parameters for shoulder, elbow and wrist, were similar for both sides (more details on kinematic findings are reported in the Supplementay Information).

### ERD/ERS

**Figure 1:**
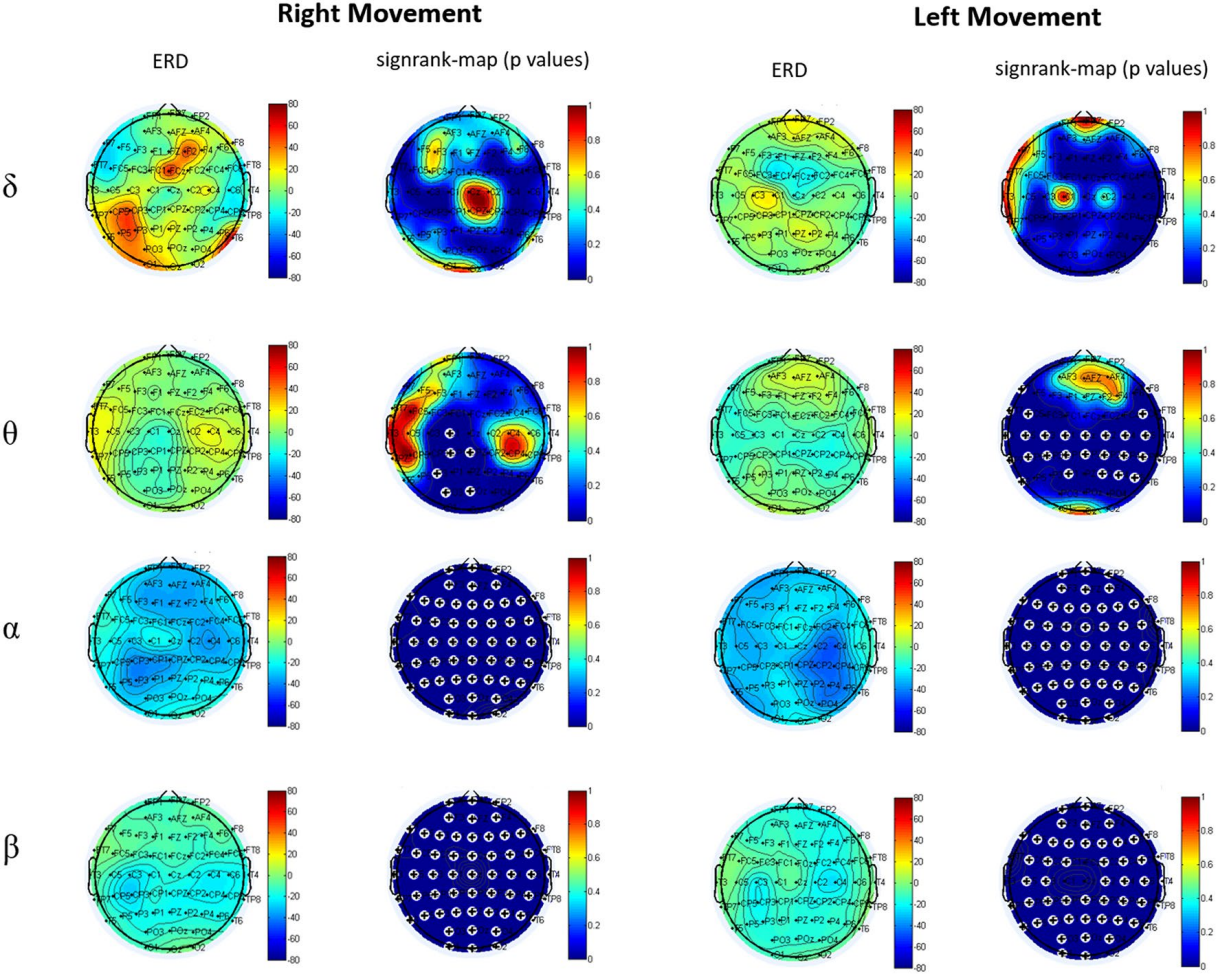
Event-related desynchronization in δ, θ, α, β bands and the relative signrank-maps comparing Movement vs. Rest. The “+” marks indicate electrodes with a significant rest-move difference (the figure was obtained with Matlab R2018 https://it.mathworks.com/products.html).

We derived the ERD/ERS maps for all frequency bands, arms and subjects and used them for group analyses (Fig. 1).

In α and β bands the mean maps revealed a widespread ERD during both right and left Mov. ERD was higher on the contralateral central and parietal regions. In θ band we found a significant ERD on the left central-parietal-occipital electrodes during the right movement and a bilateral ERD on the central-parietal regions during the left movement. No significant ERD was observed in δ band.

### Functional connectivity and network topology

**Figure 2:**
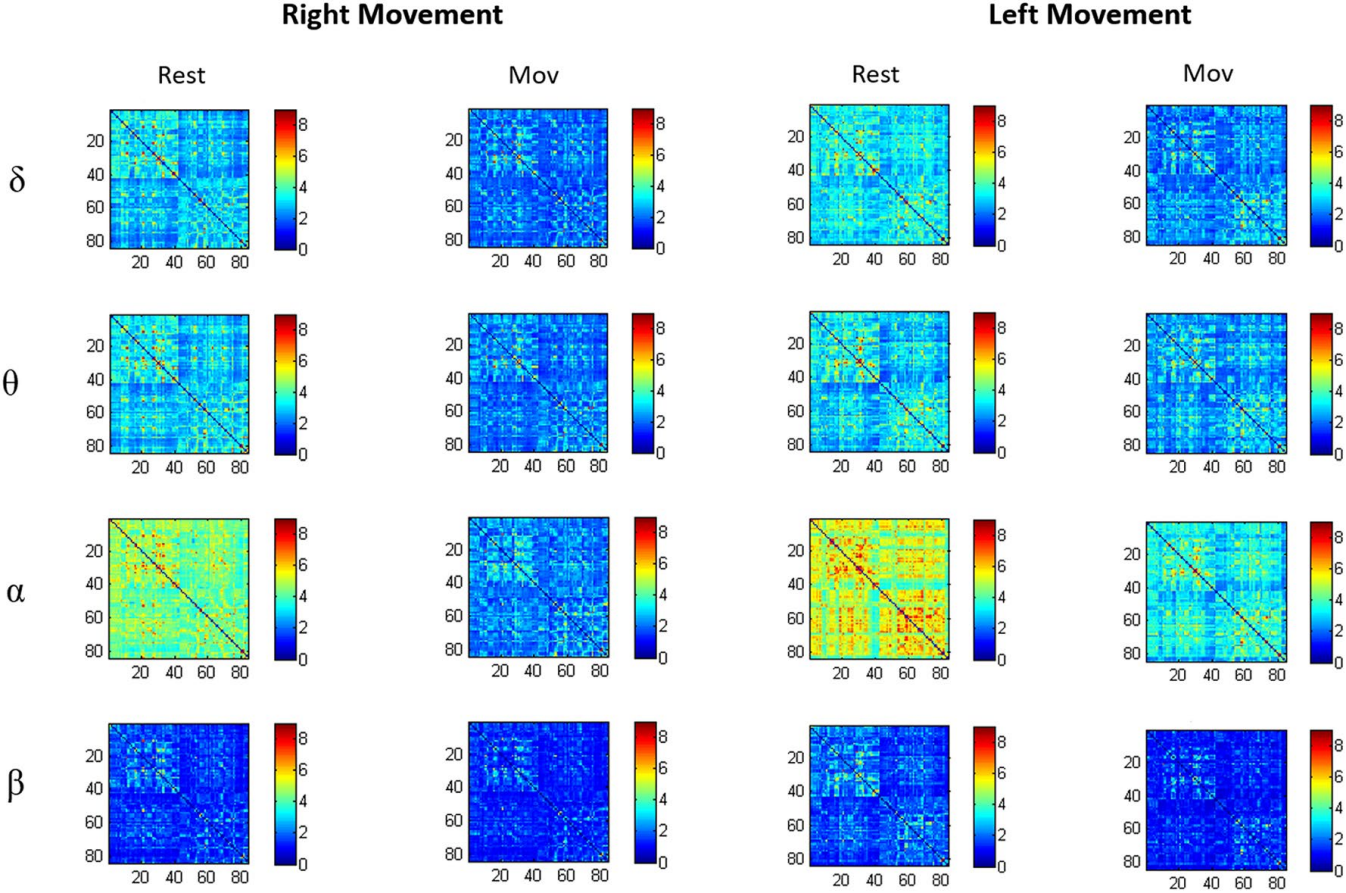
Connectivity matrices in Rest and Mov conditions averaged across subjects in δ, θ, α, β frequency bands. Each matrix element provides the color-coded LLC value between Brodmann areas as indicated by the colour bar. The upper left quadrant of the matrices depicts LLC among Brodmann’areas of the left hemisphere and the lower right quadrant shows LLC among areas of the right hemisphere (the figure was obtained with Matlab R2018 https://it.mathworks.com/products.html).

Figure 2 illustrates the connectivity matrices in the four considered frequency bands.

As can be seen on the figure, movement induces a decreased Lagged Linear Coherence (LLC) mainly in α band during both right and left Mov as compared to Rest (respectively *p* = 0.008 and *p* = 0.038). Some difference was observed in θ band for both arms (respectively *p* < 0.001 for right arm and *p* = 0.002 for left arm) and in δ band for the left movement (*p* < 0.001).

**Figure 3:**
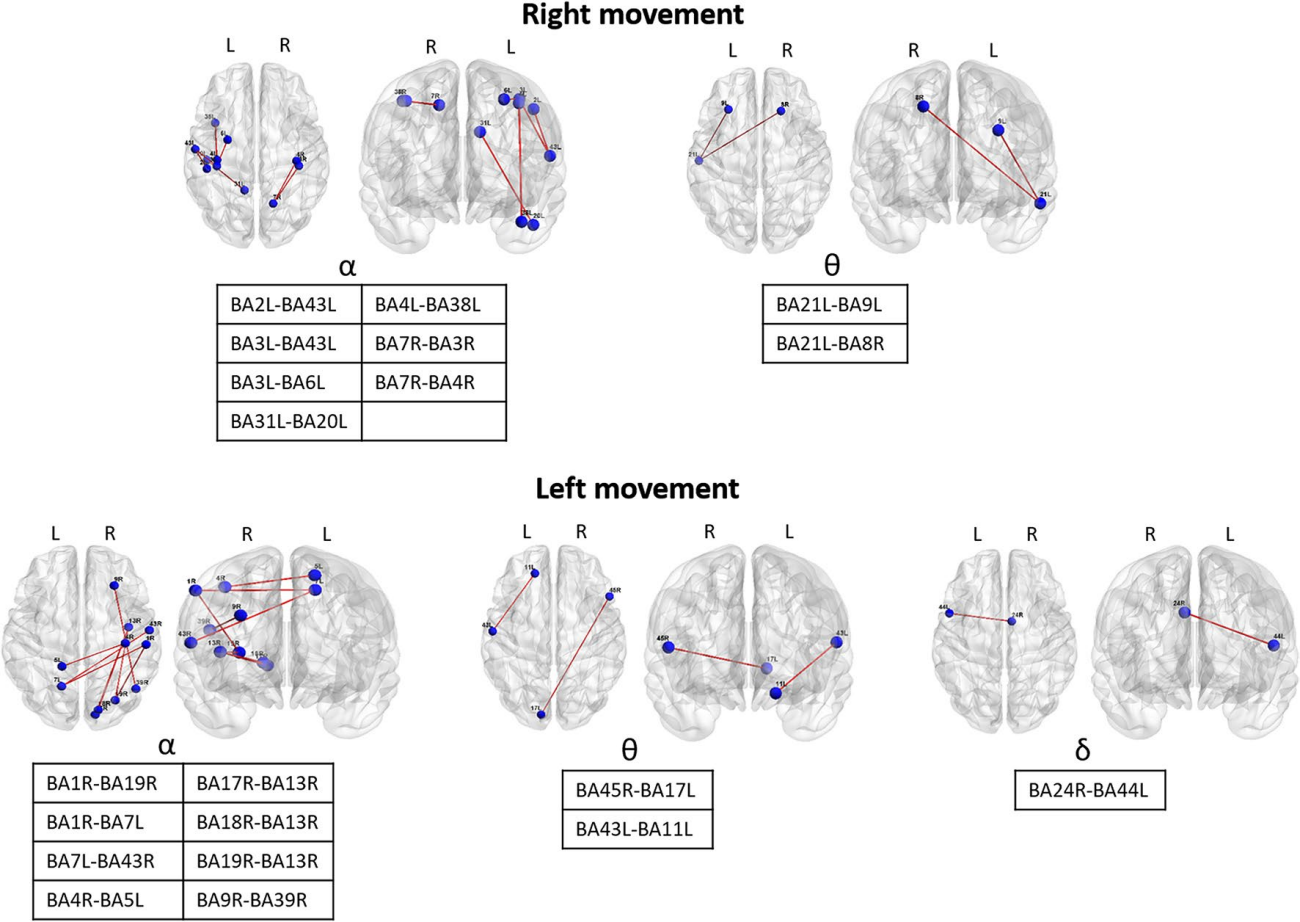
Significant FC links (LLC scores) derived from groups comparison. The figures denote the edges that reduced the strength of connectivity to each node during the movement compared to the resting condition (*p* < 0.05, corrected for multiply comparisons) (the figure was obtained with the BrainNet Viewer 1.7 http://www.nitrc.org/projects/bnv/).

Figure 3 shows the Brodmann areas with decreased LLC during the reach&grasp movement, visualized with the BrainNet Viewer (Xia et al., 2013, http://www.nitrc.org/projects/bnv/)^27^.

The right arm movement causes a LLC reduction mainly in α band among several Brodmann areas of the left hemisphere. Namely: 1) among left Brodmann areas with somatosensory function (between BA2 and BA43, between BA3 and BA43), 2) between the left primary somatosensory cortex (BA3) and the left premotor cortex (BA6), 3) between the left posterior cingulate cortex (BA31) and the left temporal lobe (BA20), 4) between the left primary motor cortex (BA4) and the left temporal lobe (BA38). Interestingly, the right superior parietal lobule (BA7) shows decreased LLC with the right primary somatosensory cortex (BA3) and the right primary motor cortex (BA4). Moreover, θ band LLC was reduced between the left BA21 and the left BA9 and between the left BA21 and the right BA8.

During the movement of the left arm, the right primary somatosensory cortex (BA1) shows reduced LLC in α band with the right BA19 and the left BA7. The left BA7 also shows reduced coherence with the right BA43. Moreover, the right primary motor cortex (BA4) shows reduced coherence with the left BA5, the right occipital cortex (BA17, BA18, BA19) with the right BA13, the right BA9 with the right BA39. θ band LLC was decreased between the right BA45 and the left BA17 and between the left BA43 and the left BA11. δ LLC was decreased between the right BA24 and the left BA44. We did not observe any change in β band for both right and left movements.

The topological parameters did not differ between Rest and Mov in different frequency bands. Tables 1 and 2 show the mean values of Cw, Lw and Sw in Rest and Mov for right and left movements and the corresponding statistical findings.

**Table 1:** Wilcoxon test for Cw, Lw and Sw between the two different experimental conditions (Rest and Mov) for each EEG frequency band during the trials performed with the right arm.

	Right arm								
	Cw			Lw			Sw		
	Rest	Mov	*p* value	Rest	Mov	*p* value	Rest	Mov	*p* value
Delta	1.03 (0.35)	1.07 (0.20)	0.285	0.89 (0.22)	0.88 (0.20)	0.878	1.36 (0.98)	1.34 (0.62)	0.575
Theta	1.04 (0.31)	1.13 (0.32)	0.386	0.86 (0.22)	0.85 (0.29)	0.878	1.41 (0.94)	1.66 (1.16)	0.575
Alpha	1.39 (0.56)	1.10 (0.35)	0.241	0.77 (0.44)	0.84 (0.25)	0.721	2.50 (1.85)	1.49 (0.84)	0.203
Beta	0.54 (0.18)	0.70 (0.24)	0.059	1.47 (0.34)	1.43 (0.48)	0.508	0.41 (0.21)	0.58 (0.35)	0.093

**Table 2:** Wilcoxon test for Cw, Lw and Sw between the two different experimental conditions (Rest and Mov) for each EEG frequency band during the trials performed with the left arm.

	Left arm								
	Cw			Lw			Sw		
	Rest	Mov	*p* value	Rest	Mov	*p* value	Rest	Mov	*p* value
Delta	0.98 (0.25)	1.00 (0.38)	0.878	0.90 (0.16)	0.94 (0.24)	0.959	1.15 (0.47)	1.22 (0.77)	0.959
Theta	0.92 (0.36)	1.08 (0.34)	0.074	0.97 (0.29)	0.88 (0.31)	0.285	1.12 (0.67)	1.48 (0.90)	0.282
Alpha	1.58 (0.46)	1.39 (0.47)	0.386	0.62 (0.20)	0.68 (0.21)	0.445	3.02 (1.78)	2.55 (0.93)	0.333
Beta	0.52 (0.18)	0.53 (0.21)	0.721	1.51 (0.33)	1.50 (0.35)	0.878	0.38 (0.23)	0.40 (0.26)	0.799

## Discussion

**Figure 4:**
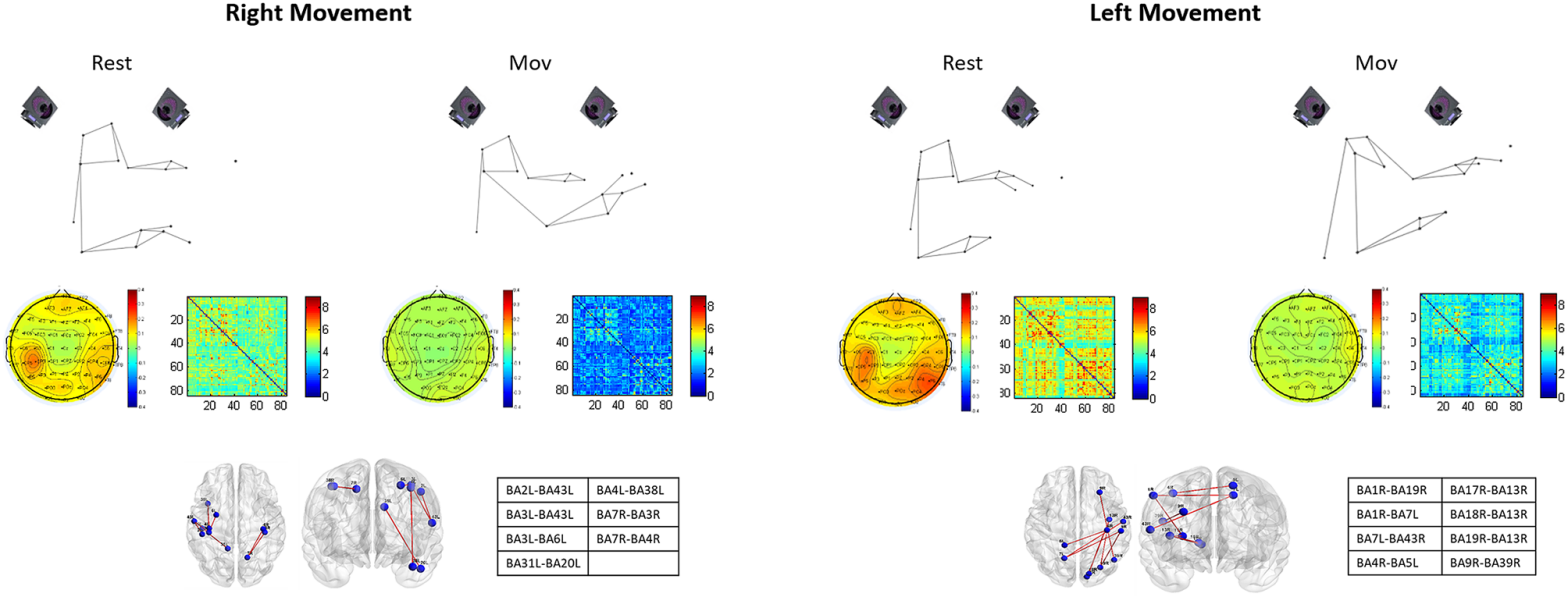
The figure sums up the main changes of cortical activity in α band induced by reach&grasp movement. In the sections A and B of the figure we show, both in Rest and Mov conditions, respectively for the right and left reach&grasp movement: (1) ERD/ERS averaged maps, (2) functional connectivity averaged matrices, (3) Brodmann areas with decreased lagged linear coherence during the reach&grasp movement.

Within the framework of an integrated method able to simultaneously measure brain connectivity and kinematics of the reach&grasp movements, we described the cortical function in terms of FC during the execution of this motor task in healthy subjects. Moreover, we demonstrated that, during the reach&grasp movement, cortical network preserves its small-world organization. Figure 4 sums up the main cortical changes induced by reach&grasp movement.

As expected, ERD/ERS evaluation showed a widespread bilateral desynchronization in α band and secondarily in β band, mainly over the somatosensory and motor areas during both the right and left movements^28,29^. In θ band we observed a different behaviour between the right and left movements, being θ activity bilaterally desynchronized on the centro-parietal electrodes during the left movement while during the right movement ERD was only observed on the contralateral centro-parietal electrodes. The coherence analysis showed very interesting results. Summing up, we noticed a coherence reduction among ROIs during the movement compared to the rest state mainly in α band for both arms. However, when analysing the pairs of ROIs with reduced coherence during the movements, we observed a different behaviour between right and left arm movements. In particular, the left arm movement was associated to the modulation of coherence among the right occipital regions, the insular cortex and the somatosensory cortex. On the other hand, we did not observe the same involvement of the occipital cortex during the right movement. This probably means that the movement performed with the non-dominant arm (left arm in our subjects) requires a higher visual control. The insular cortex has been demonstrated to be involved in different processes as touch processing^30^, integration of limb and field coordinates^31^, motor planning^32^ and error awareness^33^. In this context, it seems that the insular cortex, together with the proprioception control exerted by the somatosensory cortex^34^ and the visual control^35^, is pivotal in performing a finalistic motor task as reach&grasp with the—non-dominant arm. The hypothesis of the left movements being a less automated process than the right, dominant one is also supported by changes in FC between the right BA9 and BA39, both involved in intentional executive control of behaviour in general and movement in particular^36^. Moreover, the right BA39 is also involved in the visuospatial processing, which allows object localization^37^. Left (non-dominant) movement is associated with a complex and widespread contralateral cortical fronto-temporo-parieto-occipital network with probably a top-down control process involving executive functions. This view is in line with the concept of the “attentional landscape” which represents that attention space which computes all relevant information representing the pre requisite for properly planning and performing a complex movement^38,39^. Our observations suggest that the planning of a movement with the non-dominant side, which is more challenging for the subject, requires an attention effort and enacts an integrated cortical network which drives the motor action for obtaining a performance similar to that of the dominant side (as the kinematic measurements demonstrate). On the other hand, the right (dominant) movements do not seem to activate such a widespread contralateral network, being in this case involved the primary somatosensory cortex (areas BA2 and BA3), premotor and supplementary motor cortex (BA6), primary motor cortex (BA4), temporal lobe (BA20 and BA38) and dorsal posterior cingulate cortex (BA31). Moreover, during the right movement, left primary somatosensory cortex (BA3) shows a reduced coherence with left BA43, which has been described to be active during vibrotactile digit stimulation^40^. Another interesting finding is the involvement of the ipsilateral superior parietal lobule. Namely, during the movement with the right arm the right BA7 shows reduced coherence with the right primary motor cortex (BA4) and the right primary somatosensory cortex (BA3) while, during the movement with the left arm, the left BA7 shows reduced coherence with the right somatosensory areas (BA1 and BA43) and the left BA5 with the right primary motor cortex (BA4). In our experimental setting, which implies a whole hand grasping task, the contralateral superior parietal lobule does not show any modifications of coherence during the motor task. This finding is consistent with previous evidence suggesting that precision grip movement determines the activation of the contralateral human anterior intraparietal sulcus region (BA7) which instead is not activated during a whole hand grasping task^41^. Some evidence also suggest that the superior parietal lobule plays a role when subjects observe motor actions done by others^42,43^. In our setting, we may argue that the involvement of the ipsilateral superior parietal lobule might be related to the observation of the motor action executed by one’s own arm, but a specific experimental design is needed to better elucidate this issue.

Previous studies suggest that θ band activity is likely involved in intentional and working memory processes^44^ as well as in supervising motor tasks in order to detect and correct errors in motor performance^45^. More deeply, θ ERS was observed in the planning phase of a movement, while during the motor task execution θ activity tends to decrease^46^. We observed bilateral centro-parietal ERD during the left movement while during the right movement ERD was observed only on the contralateral centro-parietal electrodes. The ERD in θ band, defined as a power spectrum variation during the motor task as compared to the rest phase, is consistent with the time course of θ spectral perturbation, which increases before the task and decreases during the movement^46^. During the right movement, only a few ROIs show a significant modification of FC in θ activity. These ROIs are mainly involved in memory function, as BA21^47^, and executive function, namely BA21, BA9 and BA8^36,48–50^, supporting the idea of a role of θ activity in intentional processes. Indeed, during the movement with the right arm, θ band LLC was reduced between the left BA21 and the left BA9 and between the left BA21 and the right BA8. During the left movement, θ band LLC decreases between the right BA45 and the left BA17, involved in executive functions/error processing^51^ and visual attention^35^ respectively. Moreover, the left BA43 with somatosensory function^40^ showed reduced LLC with the left BA11, having decision making function^52^.

δ activity is related to decision making processes and it seems particularly relevant in selecting a specific motor strategy among co-existing alternative motor schemes^53,54^. Previous evidence suggests that δ LLC between BA44 and BA6 is enhanced during action execution^55^. In our setting, we confirmed the involvement of BA44 in motor action; however, BA44 showed a reduced coherence with the right anterior cingulate and only during the movements with the left arm.

In conclusion, we propose a multidimensional approach aimed to describe the cortical correlates of upper limb kinematics. This method might contribute to a better characterization of the neural and biomechanical processes underlying motor impairment in different clinical conditions, such as stroke. In this framework, we could further define those brain plasticity processes promoting or delaying motor recovery, tailoring specific treatments for patients with otherwise similar symptoms.

## Methods

In a previous study, the Authors recruited ten healthy subjects^21^ and found significant EEG changes during movement of upper limb compared to resting state. Here we also recruited ten healthy subjects (40.2 ± 7.4 years), which would give 80% power to detect a large effect (w = 0.90) at an α level of *p* = 0.05 (GPOWER 3.1^56^). All of them were right-handed, according to the Edinburgh questionnaire^57^. The research was approved by the local ethics committee (Fondazione Policlinico Universitario A. Gemelli, Prot N. 0007987/17) and complies with the Helsinki Declaration. Written informed consent was obtained for each subject.

### Experimental design

**Figure 5:**
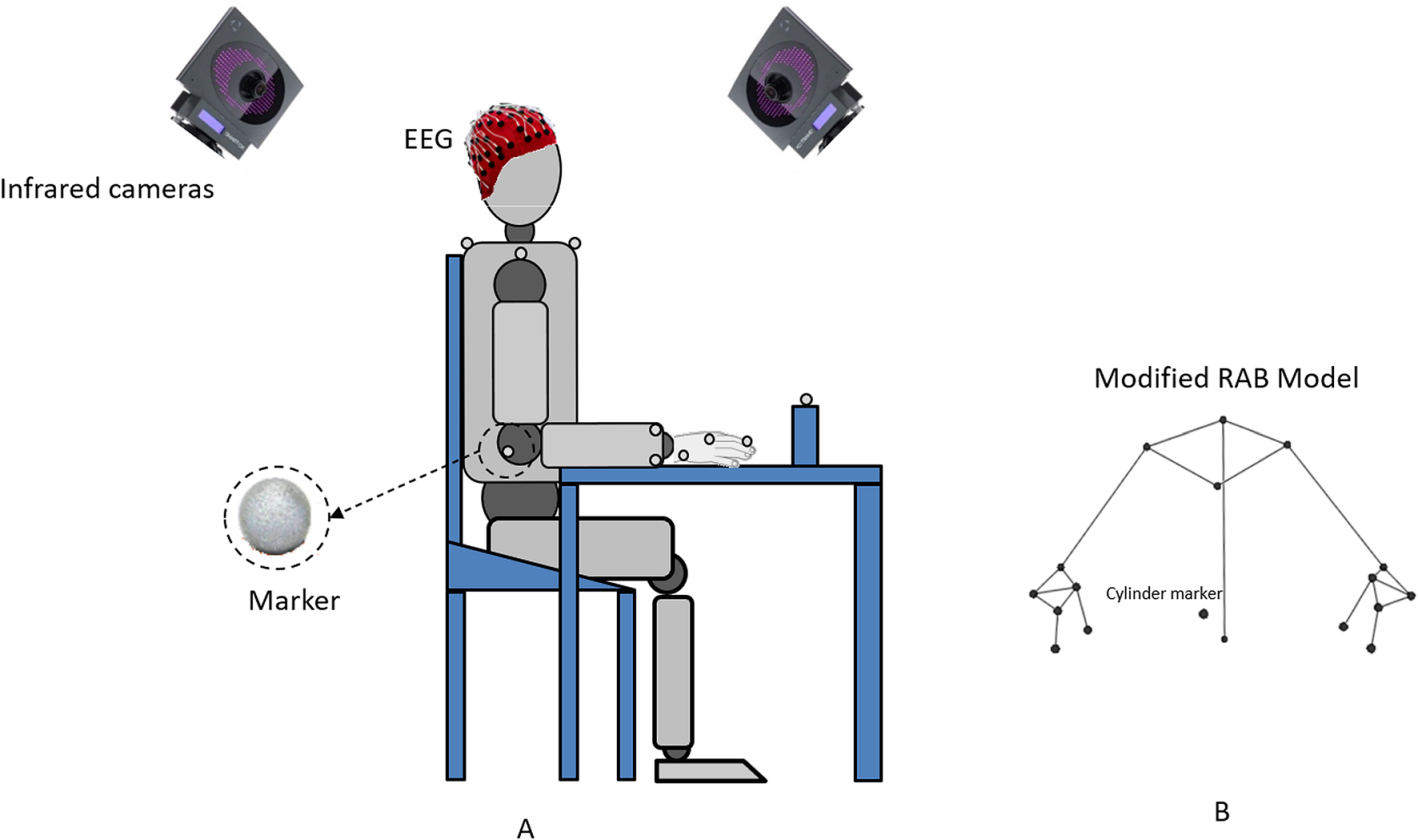
(**A**) The sagittal view of a subject in his initial position before performing the task. The spheres on the anatomical landmarks represent the passive reflective spherical markers which, in combination with infrared cameras, allow the motion capture. The subject wears an EEG cap. (**B**) Subject’s modelling according to the RAB kinematic protocol and the position of the cylinder marker.

**Figure 6:**
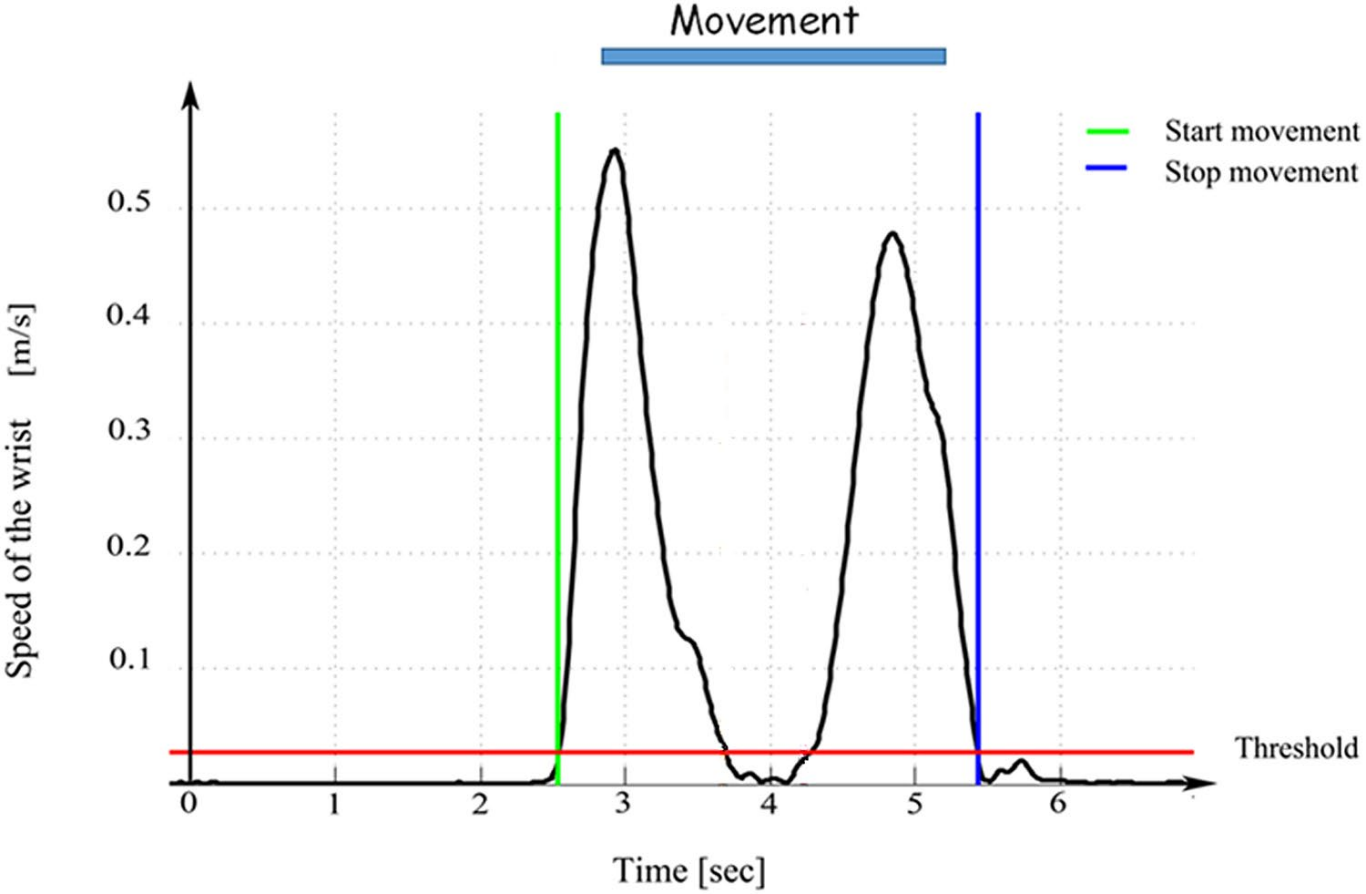
Speed of the wrist during the reach&grasp cycle. The red line indicates the 5% threshold used for marking the starting and ending timepoints of the movement.

Figure 5 shows the experimental setting. Recordings were performed in a quiet room with normal indoor temperature and lighting. In the starting position, the participants were seated comfortably at a height-adjustable table with the hips and knees flexed at 90° and both feet flat on the ground. Both arms were resting on the table so that the shoulder was in a neutral position, the elbows were flexed approximately at 90°, the forearms were pronated, and the wrists were held with the palms on the table. A cylinder (height: 12.5 cm, diameter: 5.5 cm) was placed at 75% of the participant’s maximum reach distance. From the initial position, each participant was instructed to reach forward and grasp the cylinder, release the cylinder, and return his/her arm to the initial position. The participants were instructed to perform 15 self-paced movements with both hands. The first section of the experiment consisted in 15 movements performed with the right dominant arm and the second one in 15 movements with the left arm. Each task was simultaneously assessed by an optoelectronic system equipped with 8 infrared cameras synchronized with a 64-channels high density EEG (NEURO PRAX EEG, neuroConn). A trigger box device adopting as trigger signal a square wave with 5-V amplitude guaranteed synchronization. The trigger box was connected to the optoelectronic system SMART-DX 500 (BTS, Milan, Italy) by a USB port and to the EEG equipment by a BNC connector. By the trigger box we started the motion capture recording and the trigger signal simultaneously appeared on the EEG recording. Kinematic parameters were measured by placing 17 markers on anatomical landmarks in accordance with a validated upper limb and trunk biomechanical model (RAB model—modified) and another marker on the cylinder (kinematic measurements are extensively described in the Supplementary Information section). We used kinematic recordings to define the onset and the end of the movement. Briefly, we identified the onset of the movement as the first instant when the module of the wrist velocity exceeded 5% of the peak-velocity of reaching. The end of the cycle was detected as a decrease in wrist marker velocity to less than 5% of the maximum velocity during the returning of the wrist to the initial position after grasping^58,59^ (Fig. 6). Synchronization across systems allowed us to identify the time points corresponding to the start and the end of each movement on the EEG recordings. The jerk was used in order to evaluate the motor performance in terms of smoothness^60–62^, that is defined as the time derivative of marker acceleration (details on the jerk calculation are reported in the Supplementary Information section). Low values of jerk indicate an increase in movement smoothness.

EEG was recorded by placing Fpz, Cz, Oz, and the preauricular points according to the international 10/20 system. EEG data were continuously recorded during the two sections at a sampling rate of 512 Hz. Event-markers, defined accordingly to the kinematic profiles, allowed to identify movement-related segments (Mov) on EEG signals. The epoch of 2 s before each movement was considered as baseline period (Rest).

### EEG data analysis

Data were pre-processed in Matlab R2018 (Math-Works, Natick, MA) using scripts based on EEGLAB 12 (http://www.sccn.ucsd.edu/eeglab). The EEG recordings were band-pass filtered from 1 to 30 Hz in order to suppress muscle artefacts, since muscular contraction shows a frequency greater than 30 Hz^63^. Visible artefacts (i.e. eye movements, cardiac activity, and scalp muscle contraction) were removed using independent component analysis (ICA)^64^, and RELICA algorithm, with an index retention threshold of 85%, was used to exclude non-brain signals from the subsequent analysis^65^. The event related synchronization/desynchronization (ERS/ERD) measures were used to quantify the movement-related changes of brain activity in δ, θ, α and β frequency bands (δ: 1–4 Hz, θ: 4–8 Hz, α: 8–13 Hz, β: 13–30 Hz). ERD/ERS values were defined as the percentage decrease/increase of the power spectral density (PSD, μV^2^/Hz) during Mov with respect to the Rest as follows:

1 $$ERD^{\mu } = \frac{{PSD_{task}^{\mu } - PSD_{rest}^{\mu } }}{{PSD_{rest}^{\mu } }}$$

where the symbol µ indicates respectively δ, θ, α and β frequency bands. A mean ERD was estimated for each subject and then for the entire sample. Accordingly, event-related PSD decrements, representing a decrease in synchronization of the underlying neuronal populations and indicating cortical activation, resulted in negative values.

### EEG source imaging and functional connectivity

The functional brain connectivity was estimated using lagged linear connectivity at the source space, which does not suffer for volume conduction. EEG data were processed using standard low-resolution brain electromagnetic tomography (sLORETA) software for localizing the cortical sources http://www.uzh.ch/keyinst/NewLORETA/LORETA01.htm)^66^. The EEG cross-spectra and the corresponding 3D-cortical distribution of the electric neuronal generators were computed for each frequency band, under the Rest and Mov conditions and for each subject separately^67,68^. The solution space was restricted to the cortical gray matter and the Montreal Neurologic Institute average MRI brain (MNI)^69^ was used as a realistic head model.

We calculated lagged linear connectivity, i.e. excluding coherences with zero phase lag^70^, for each Rest and Mov epoch in the same frequency ranges and used a whole-brain Brodmann areas (BAs) atlas for selecting the 42 BAs in each hemisphere as regions of interests (ROIs) for performing the FC analysis between pairs of ROIs.

The Lagged Linear Coherence (LLC) in a specific frequency band ω is defined by the following equation:

2 $$\rho_{xy}^{2} (\omega ) = \frac{{[{\text{Im}} (s_{xy} (\omega ))]^{2} }}{{s_{xx} (\omega )s_{yy} (\omega ) - [{\text{Re}} (s_{xy} (\omega ))]^{2} }}$$

where $s_{xx}$ and $s_{yy}$ are the power spectral densities of two source signals x and y respectively, $s_{xy}$ is the cross power spectral density of x and y, and ω is the reference frequency band^70^.

Lagged Linear Coherence was used to calculate the connectivity matrices for each subject in Rest and Mov for all bands. The connectivity matrices of all subjects in the Rest and Mov were processed in Matlab R2018 calculating with specific functions the average connectivity matrix for each condition and arm. In order to avoid the issue of arbitrary thresholding, our network analyses were conducted on fully connected graphs^21^ and the corresponding lagged coherence values were used as the weight of the edge connecting each pair of ROIs (nodes)^71^.

### Graph analysis

For each weighted connection matrix, MATLAB scripts available from The Brain Connectivity Toolbox (BCT) (https://sites.google.com/site/bctnet/) were used to compute network metrics that summarize global architecture. The topological attributes of the brain networks were examined into different levels, calculating both local and global metrics for each FC measure. We calculated mean weighted clustering coefficient (Cw) and mean weighted characteristic path length (Lw) as a measure of segregation and integration of the network, respectively. Cw was calculated by averaging the weighted clustering coefficient of all single nodes in the network, while Lw indicates the average of the shortest weighted paths connecting each node to all the other ones^24,72^. For each subject, the values of Cw and Lw of each band were normalized to the global mean value obtained after having averaged each such parameter through the four bands (δ, θ, α, β).

The functional integration of the network was estimated by its “small-world-ness” as a quotient between Cw and Lw normalized to the Cw_rand_ and Lw_rand_ of an equivalent weighted random network^24,72^ as decribed in the following equation:

3 $$Sw = \frac{{Cw/Cw_{rand} }}{{Lw/Lw_{rand} }}.$$

This is a scaled measure indicating the small-world-ness characteristic of the network when the quotient is higher than 1. In that case, the Lw is similar to that of a random equivalent graph and/or the network’s Cw is larger than that of the equivalent random graph. Finally, the Cw, Lw and Sw were averaged across subjects obtaining a mean value for each condition (respectively Rest and Mov).

### Statistical assessment

We performed a paired sample two-sided Wilcoxon signed rank test (W-test) to compare the Jerk’s values between right and left arm. Moreover, we performed the same statistical test to identify significant differences between the PSD in the Rest and Mov, respectively. Then, we computed two-dimensional singnrank-maps of PSD from the p values to detect the topographical distribution of the significance.

We calculated connectivity contrast maps through multiple ROI-by-ROI comparisons using the Wilcoxon Signed-Rank Test (W-test) to verify whether statistical differences between Rest and Mov were present. A non-parametric approach (statistical non-parametric mapping [SnPM]) via randomization was used (5,000 permutations). Using this randomization strategy, the critical probability threshold values for the actually observed *t*-values was determined with correction for multiple comparisons across all voxels and all frequencies. We plotted the significant connections (*p* < 0.05) onto a MRI template. Then, the Wilcoxon test at significance level *p* < 0.05 was used to detect the differences in the corresponding topological parameters (Cw, Lw and Sw) between Rest and Mov.

## Supplementary Information

Supplementary Information.

## Data Availability

The data that support the findings of this study are available from the corresponding author upon reasonable request.
