# Supplementary material for: Connectivity modulations induced by reach&grasp movements: a multidimensional approach
Source: Sci Rep. 2021 Nov 29;11:23097. doi: 10.1038/s41598-021-02458-x (PMC8630117; doi:10.1038/s41598-021-02458-x)
Supplement: Supplementary file 1 — Supplementary Information. [file 41598_2021_2458_MOESM1_ESM.pdf]

TITLE

Connectivity modulations induced by reach&grasp movements: a multidimensional approach

AUTHORS

Pietro Caliandro[1], Gloria Menegaz[2], Chiara Iacovelli[3,*], Carmela Conte[4], Giuseppe Reale[5], Paolo Calabresi[6], and Silvia F. Storti[2]

[1]UOC Neurologia - Dipartimento Scienze dell'Invecchiamento, Neurologiche, Ortopediche e della Testa-Collo, Fondazione Policlinico Universitario A. Gemelli IRCCS, Rome, Italy – pietro.caliandro@policlinicogemelli.it

[2]Dept. of Computer Science, University of Verona, Strada Le Grazie 15, 37134, Verona, Italy – gloria.menegaz@gmail.com; silviafrancesca.storti@univr.it

[3]UOC Riabilitazione e Medicina Fisica - Dipartimento Scienze dell'Invecchiamento, Neurologiche, Ortopediche e della Testa-Collo, Fondazione Policlinico Universitario A. Gemelli IRCCS, Rome, Italy – chiara.iacovelli@policlinicogemelli.it

[4]IRCCS Fondazione Don Carlo Gnocchi, Florence, Italy – cconte@dongnocchi.it

[5]UOC Neuroriabilitazione ad Alta Intensità - Dipartimento Scienze dell'Invecchiamento, Neurologiche, Ortopediche e della Testa-Collo, Fondazione Policlinico Universitario A. Gemelli IRCCS, Roma, Italy – giuseppe.reale@policlinicogemelli.it

[6]Clinica Neurologica, Dipartimento di Neuroscienze, Fondazione Policlinico Universitario Agostino Gemelli IRCCS, Università Cattolica del Sacro Cuore, Roma, Italy – paolo.calabresi@policlinicogemelli.it

APPENDIX

Kinematic measurements

Movement Analysis

Kinematic recordings

Recordings were performed using the optoelectronic motion analysis system (SMART-DX 500, BTS, Milan, Italy). This system consists of eight infrared cameras operating at a sampling rate of 100 Hz which detect the motion of passive reflective spherical markers (15 mm in diameter). We used a biomechanical model consisting of four segments (hand, forearm, arm, and trunk) as previously described [1]. In detail Specifically, we placed 17 markers over the right and left acromions, on the cutaneous projections of the spinous processes of the seventh cervical vertebra, on the mid sternal line between the claviculas, over the sacrum, and bilaterally over the olecranon, over the radial and ulnar styloid, over the head of the second metacarpal bone, over the first finger and over the second finger. Moreover, we placed another marker on the target cylinder. During the movement's execution, we acquired three-dimensional trajectories for each marker using a three-dimensional (3-D) acquisition software (Smart Capture, BTS, Milan, Italy) and a frame-by-frame tracking system (Tracklab – BTS, Milan, Italy). All the data were processed using 3-D processing tool (SMART Analyzer, BTS, Milan, Italy) and MATLAB (MATLAB R2018, MathWorks, Natick, MA, USA).

Kinematic measurements

The joint kinematics of the shoulder, elbow and wrist during the reach&grasp movements were calculated. In particular, we computed the shoulder abduction-adduction, shoulder flexion-extension, shoulder intra-extrarotation, elbow flexion-extension and wrist flexion-extension. Each joint motion curve was time-normalized to 100% of the reach&grasp cycle. Reach&grasp cycles were defined using a threshold derived from the radio and ulna markers. First, we computed the coordinates of the medial point between the radio and ulna markers as wrist coordinates, then we calculated the speed of the wrist as a derivative of the segment position. Three-dimensional coordinates of the wrist were filtered with a self-adapting low-pass filter resulting in a cutoff frequency of 6Hz [2]. We defined the onset of the movement from the start position as the first instant when the module of the velocity exceeded 5% of the peak-velocity of reaching. The end of the cycle was detected as a decrease in wrist marker velocity to less than 5% of the maximum velocity during the returning of the wrist to the initial position after grasping [3,4]. Synchronization across systems allowed us to identify the time points corresponding to the start and the end of each motor movement on the EEG recordings.
The jerk was used in order to evaluate the motor performance in terms of smoothness [5-7], that is defined as the time derivative of marker acceleration. Low values of jerk indicate an increase in movement smoothness. Normalized jerk can be determined with the following formula:

$$Jerk = \sqrt{\frac{1}{2} \cdot \int_{t1}^{t2} \left[\left(\frac{d^3x}{dt^3} \right)^2 + \left(\frac{d^3y}{dt^3} \right)^2 + \left(\frac{d^3z}{dt^3} \right)^2 dt \cdot \frac{(t2-t1)^5}{L^2} \right]} \quad (1)$$

The jerk was calculated from wrist coordinates recorded by the optoelectronic system, t1 represents the onset of the movement, t2 the end of the movement, $\frac{d^3x}{dt^3};\frac{d^3y}{dt^3};\frac{d^3z}{dt^3}$ are the third order partial derivatives of the 3D coordinates x, y, z and L is the extension of the trajectory.

Kinematics Results

The jerk value was 703.63±183.39 for the right arm and of 641.86±194.54 for the left arm, respectively. Figures S1a and b show the kinematic parameters for shoulder, elbow and wrist, for the right and left side. In our setting, the shoulder is bilaterally abducted, flexed and extra-rotated. During the first 10-15% of the Mov period, shoulder abduction is about 30° and then it begins to reduce at 15-20% of the cycle. Between 40% and 60% of the Mov period, the shoulder reaches a plateau with an abduction of about 15° and starting from the 60% of Mov period the curve rises and reaches an abduction of 40° in the last 10% of the movement. On the sagittal plane, the shoulder flexion begins to increase soon after the movement onset and reaches a plateau of flexion of about 70° between the 20% and 70% of the movement period, and shortly after that the curve of flexion begins to descend down to a flexion of about 10°. On the transverse plane the shoulder extra-rotation begins to increase soon after the beginning of the movement, reaches its plateau between 20% and 70% of the movement with an extra-rotation of about 50° and then it begins to reduce. On the sagittal plane, the elbow is flexed at 90° (-90° of extension in Figures S1a and b) for the first 10% of the Mov period, then it progressively reaches a

plateau of extension (about 140°) between 30% and 60% of Mov and returns to the initial position. The wrist is bilaterally in extension between 10-15% and 80% of Mov. In the first 10-15% and in the last 20% of Mov, the kinematics of the wrist is different between the two arms, with the right wrist tending to flexion and the left wrist tending to a more neutral position.

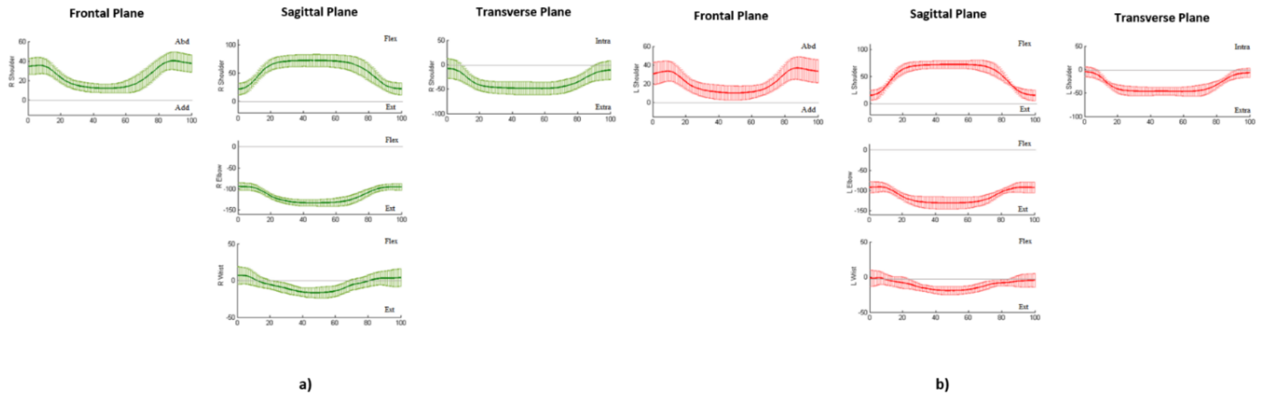

Figure S1. Kinematics findings of the right (a) and left arm (b).

REFERENCES

1. Rab, G., Petuskey, K. & Bagley, A. A method for determination of upper extremity kinematics. in *Gait and Posture* vol. 15 113–119, https://doi.org/10.1016/S0966-6362(01)00155-2 (Gait Posture, 2002).
2. D'Amico, M. & Ferrigno, G. Technique for the evaluation of derivatives from noisy biomechanical displacement data using a model-based bandwidth-selection procedure. *Med. Biol. Eng. Comput.* **28**, 407–415, 407–415. https://doi.org/10.1007/BF02441963 (1990).
3. Coluccini, M., Maini, E. S., Martelloni, C., Sgandurra, G. & Cioni, G. Kinematic characterization of functional reach to grasp in normal and in motor disabled children. *Gait Posture* **25**, 493–501, https://doi.org/10.1016/j.gaitpost.2006.12.015 (2007).
4. Van Der Heide, J. C., Fock, J. M., Otten, B., Stremmelaar, E. & Hadders-Algra, M. Kinematic characteristics of reaching movements in preterm children with cerebral palsy. *Pediatr. Res.* **57**, 883–889, https://doi.org/10.1203/01.PDR.0000157771.20683.14 (2005).
5. Kitazawa, S. Optimization of goal-directed movements in the cerebellum: A random walk hypothesis. *Neurosci. Res.* **43**, 289–294, https://doi.org/10.1016/S0168-0102(02)00058-5 (2002).
6. Yan, J. H. Effects of aging on linear and curvilinear aiming arm movements. *Exp. Aging Res.* **26**, 393–407, https://doi.org/10.1080/036107300750015778 (2000).
7. Ceccarini, F. & Castiello, U. The grasping side of post-error slowing. *Cognition*. **179**, 1-13, 10.1016/j.cognition.2018.05.026. (2018).
